# Transcriptome analysis of muskrat scented glands degeneration mechanism

**DOI:** 10.1371/journal.pone.0176935

**Published:** 2017-05-04

**Authors:** Yimeng Li, Tianxiang Zhang, Juntong Zhou, Shuang Yang, Mengyuan Fan, Xiaoning Sun, Meishan Zhang, Shanghua Xu, Muha Cha, Xiaolong Hu, Lei Qi, Shaobi Lin, Shuqiang Liu, Defu Hu

**Affiliations:** 1 College of Nature Conservation, Beijing Forestry University, Beijing, People’s Republic of China; 2 Research Department, Zhangzhou Pien Tze Huang Pharmaceutical Co., Ltd, Zhangzhou City, Fujian Province, People’s Republic of China; International Centre for Genetic Engineering and Biotechnology, ITALY

## Abstract

The scented gland, a musk-secreting organ of male muskrats, shows clear seasonal changes. When entering the secreting season in March, scented glands gradually increase in size and active secretion starts. In September, scented glands become gradually smaller and secretion decreases. By November, scented glands are gradually replaced by adipose tissue. In this study, six healthy adult male muskrats were analysed: three from the secreting season (March) and three from the non-secreting season (November). Using RNA-Seq analysis, gene expression profiles of scented glands from both seasons were determined. Using the Kyoto Encyclopedia of Genes and Genomes (KEGG) database, we found that genes involved in calcium and TGF-beta signalling pathways were significantly more expressed in the non-secreting than in the secreting season. These changes in gene expression correlated with alterations in scented gland size. Both calcium and TGF-beta signalling pathways are important regulators of cell apoptosis, which may thus be involved in muskrat scented gland degeneration.

## Introduction

The muskrat (*Ondatra zibethicus L*.), also known as green root mink or water rat, is a medium-sized, semi-aquatic herbivore, valued for its medicinal applications and fur. It belongs to the order Rodentia and the family Cricetidae. Male muskrats develop a specialised organ, the scented gland, in the lower abdomen between the muscles and skin. During the breeding season, from March to October, the scented glands of adult male muskrats can produce a substance referred by Van Dorp et al [1] as American musk. The main components of the muskrat musk are similar to those in natural musk, such as cyclic ketones, alkanes, esters, aldehydes, and organic acids [2–4]. They possess anti-inflammatory and analgesic properties [5–6], they enhance cardiac function, improve myocardial ischemia, and protect the heart from ischemic injury [7–8].

Muskrat scented glands morphology exhibits clear seasonal changes, which can be divided in four stages: non-secreting, scented gland development, peak-level secretion, and continuous secretion [9]. In March, the secretion stage begins and the scented glands start to develop, achieving peak size in April, and then undergoing further changes until August. The diameter of a scent-filled scented gland can reach 1.6 cm, with a length of up to 3.7 cm and a weight of approximately 3.0 g [10]. At the beginning of September, the glands start to atrophy, they shrink near the opening of the penis to the size of a soybean, and by November are gradually replaced by adipose tissue. We speculate that these dramatic changes must be genetically regulated. Therefore, in the current study we employed transcriptome analysis to identify pathways involved in regulating the degeneration of scented glands.

Cell apoptosis is a genetically controlled, autonomous, orderly, and active death process. Currently, there are three known apoptotic pathways: mitochondrial (also known as endogenous), death receptor, and endoplasmic reticulum (ER). Studies in recent years have suggested that mitochondria are also the main regulatory site for most cell apoptotic events. The endogenous apoptotic pathway can be induced by a variety of factors, such as cells located away from their original growth environment, loss of growth factors or supporting hormones required for survival, and DNA damage [11]. The death receptor pathway is induced by extracellular signals and is thus known as the exogenous apoptotic pathway. The ER, as a pivotal platform for signal transduction, plays an important role in the apoptotic process. Hence, the recently discovered ER pathway is also referred to as ER stress (ERS) induced apoptosis.

The transcriptome encompasses all RNA expressed under a specific condition. It is employed to identify changes in gene expression under different conditions and reveals the interaction between different genes and their respective functions [12]. Transcriptome sequencing can quickly and fully reveal the gene expression profile in specific cells or tissues under a particular condition, which is then used to study gene structure and function, alternative splicing, and the prediction of new transcripts [13–17]. Although muskrats are of high economic and medicinal value, research on their molecular biology is limited and gene database resources are scarce. This study applied transcriptome sequencing technology combined with morphological changes of the scented glands to elucidate pathways regulating scented gland cell apoptosis. The findings provide the basis for understanding seasonal changes of muskrat scented glands and lay the foundation for further studies on the gene regulatory processes involved.

## Materials and methods

### Animals and sample collection

Six healthy adult male muskrats of similar size and weight (average weight of 1.5 kg) were purchased from Jinmu Technologies (Xinji City, Hebei, China): three were from the secreting season (March) and three were from the non-secreting season (November). Scented gland tissues were collected, cryopreserved in liquid nitrogen, and transferred to the laboratory for subsequent transcriptome analysis. All muskrats were put to death by dislocation after anesthesia, the anaesthesia Xylazine hydrochloride, intramuscular injection to each experimental muskrat of 1.2 ml (0.8–1.2 ml/kg).

All animals were treated in accordance with the National Animal Welfare Legislation. All experimental procedures were carried out in accordance with the guidelines established by the Beijing Forestry University. And the studies were approved by Beijing Forestry University on animal care.

### Transcriptome sequencing

Total RNA of muskrat scented gland tissues was isolated using Trizol reagent (Qiagen, Valencia, CA, USA) following the manufacturer’s recommendations. Total RNA integrity was assessed using an Agilent 2100 Bioanalyzer (Agilent Technologies, Inc., Santa Clara, CA, USA) and 1% agarose gel electrophoresis. The construction of the libraries and the RNA-Seq was performed by the Biomarker Biotechnology Corporation (Beijing, China). According to the Illumina manufacturer's protocols, Oligo(dT)-magnetic beads were used to enrich the mRNA, which was then broken into short fragments. Random hexamers were employed to synthesise first-strand cDNA, using mRNA as a template. Buffer, dNTPs, RNase H, and DNA polymerase I were added to synthesise second-strand cDNA. Next to the purification of the double stranded cDNA with AMPure XP beads, the end-repairing, Poly-A tailing and, sequencing adapters linking processes were completed. The concentration and insert size of cDNA library were detected using Qubit 2.0 and Agilent 2100, and quantified with q-PCR. Finally, Paired-end sequencing was conducted on an Illumina HiSeq 2500 platform (Illumina Inc., San Diego, CA, USA), which generated approximately 100 bp Paired-End (PE) reads.

In order to obtain the clean data, the raw reads were initially processed for removing the adapter sequences and low-quality bases. Then, the Q30 and GC-content were used to assess the sequencing quality. Finally, transcriptome *de novo* assembly was performed using the Trinity program with its default parameter values [18].

The unigenes were annotated using BLAST (Basic Local Alignment Search Tool) [19] searches against the NR (Non-Redundant protein) database[20], Swiss-Prot[21], KEGG (the Kyoto Encyclopedia of Genes and Genomes) [22] and GO (Gene Ontology) [23], COG (Clusters of Orthologous Groups) [24] and KOG (Eukaryotic Orthologous Groups) [25] with E-value of 10^−5^ and HMMER software[26] with E-value of 10^−10^. The reads of the samples were compared with the transcript using Bowtie software. The comparison information was used in RSEM software to evaluate the expression level of the unigenes. The result was presented as FPKM (fragments per kilobase of transcript per million mapped reads) value.

### Reverse transcription PCR (RT-PCR)

First-strand cDNA was synthesised from total RNA using genomic DNA (gDNA) and the FastQuant RT Enzyme of the FastQuant RT Kit (with gDNA) (Tiangen, Beijing, China). The reaction mixture (20 μL) contained total RNA (260 ng), 5 × gDNA Buffer (2 μL), 10 × Fast RT Buffer (2 μ), RT Enzyme Mix (1 μL), and FQ-RT Primer Mix (2 μL). The primer mix (25 μL) contained first-strand cDNA (6 μL), 1 μL of each primer, 2 × Taq PCR MasterMix (12.5 μL), and ddH_2_O (4.5 μL) (Tiangen). The PCR primers were listed in S1 File. Amplification was carried out under the following conditions: 94°C for 3 min for the initial denaturation of the RNA/cDNA hybrid; 33 cycles at 94°C for 30 s, 55°C for 20 s, and 72°C for 20 s; plus a final extension of 5 min at 72°C. The glyceraldehydes 3-phosphate dehydrogenase cDNA fragment was amplified by intron-spanning primers 5′-tttggcatcgtggaagga-3′ (bases 508–525) and 5′-cgaaggtagaagagtgggagt-3′ (bases 892–872). The PCR product was electrophoresed on a 1% agarose gel and individual bands were visualised by ethidium bromide staining. All experiment was repeated three times.

### Statistical analysis

Results are presented as means + standard error of the mean (SEM) or standard deviation (SD). Student’s *t*-test was used for data analysis. P value < 0.05 was considered statistically significant. In differential expression gene selection, we use FDR (False Discovery Rate)<0.01 and Fold change≥2 as a standard, FDR is obtained using the Benjamini-Hochberg method to correcting the P value.

## Results

### Transcriptome sequencing results

#### Statistical analysis of contig assembly

**Fig 1:**
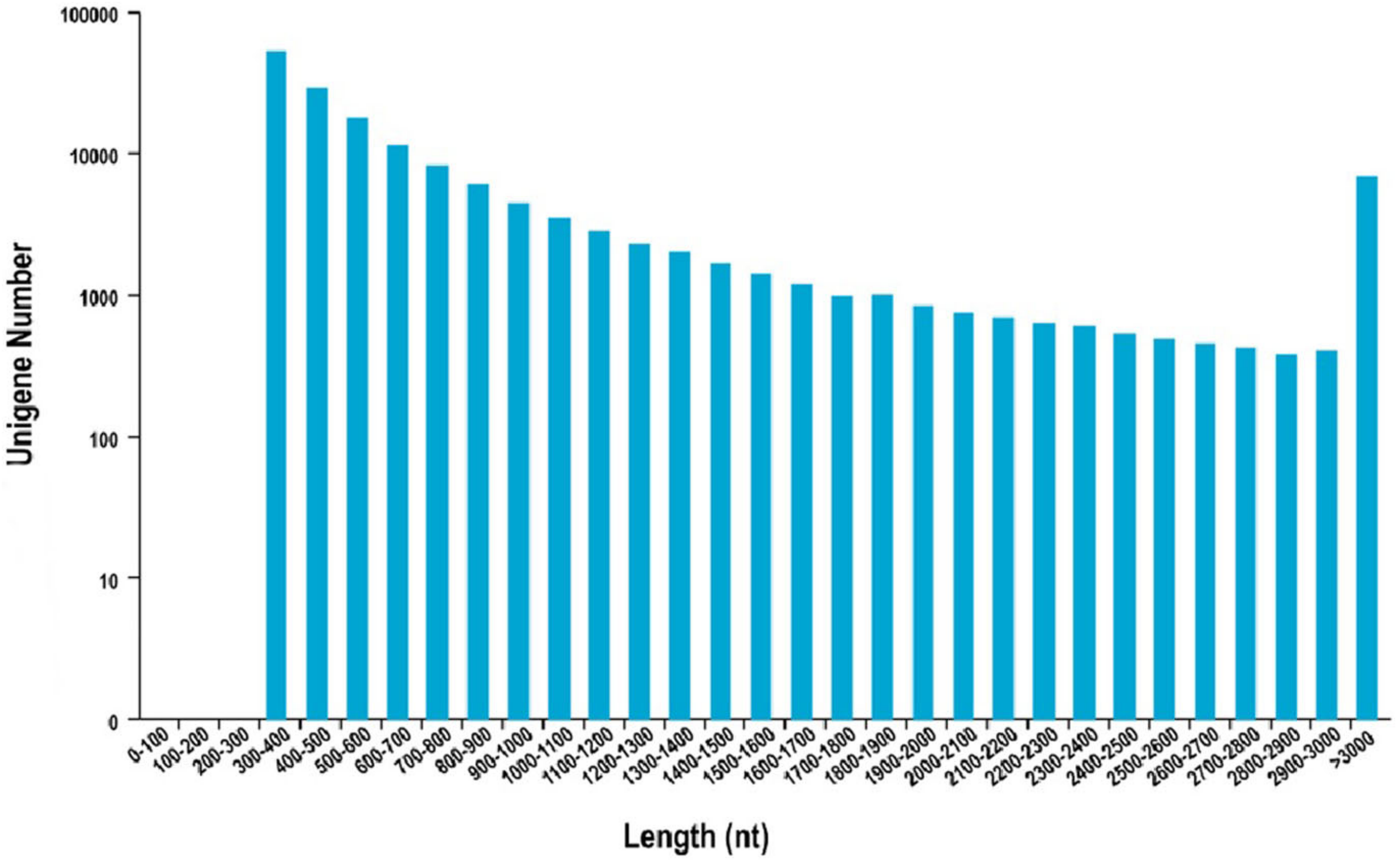
Unigene length distribution. Length of the contigs (X-axis) versus number of Unigenes obtained (Y-axis).

The Illumina HiSeq 2500 high-throughput sequencing system was adopted to sequence the transcriptome of six samples of muskrat scented glands, generating 27.84 Gb of Clean Data. Each sample produced 3.57 Gb of Clean Data, of which Q30 bases accounted for at least 88.98%. Clean reads were assembled into a total of 14,917,239 contigs. As the length increased, the number of contigs decreased accordingly. There were 14,734,073 contigs with a length of 200–300 nt, accounting for 98.77% of the total; 92,244 contigs with a length of 300–500 nt (0.62% of the total); 56,348 contigs with a length of 500–1,000 nt (0.38% of the total); 21,652 contigs with a length of 1,000–2,000 nt (0.15% of the total); and 12,922 contigs longer than 2,000 nt (0.09% of the total). After *de novo* assembly, a total of 369,675 transcripts and 157,799 Unigenes were obtained; their N50 values were 4,082 and 1,115, respectively. Among them, there were 80,933 Unigenes with a length of 300–500 nt, accounting for 51.29% of the total; 47,280 Unigenes with a length of 500–1,000 nt (29.96% of the total); and 29,586 Unigenes longer than 1,000 nt (18.75% of the total). Detailed statistical results are presented in Fig 1 and Table 1.

**Table 1:** Statistical analysis of contig assembly.

Length range	Contig	Transcript	Unigene
200–300	14,734,073 (98.77%)*	-	-
300–500	92,244 (0.62%)	99,539 (26.93%)	80,933 (51.29%)
500–1,000	56,348 (0.38%)	75,637 (20.46%)	47,280 (29.96%)
1,000–2,000	21,652 (0.15%)	56,967 (15.41%)	17,531 (11.11%)
2,000+	12,922 (0.09%)	137,532 (37.20%)	12,055 (7.64%)
Total number	14,917,239	369,675	157,799
Total length	775,299,497	795,083,030	134,010,164
N50 length	48	4,082	1,115
Mean length	51.97	2,150.76	849.25

Values indicate the number (and corresponding percentage in parenthesis) of contigs, transcripts, and Unigenes within each length range.

*denotes the number and percentage of contigs within the 0–300 nt length range.

Additionally, the total number, total length, N50 length and average (mean) length of contigs, transcripts, and Unigenes are reported.

#### Functional annotation of Unigenes

Database comparison provided functional annotation for All-unigenes. We compared all of the unigenes to the NCBI nr database using BLAST with an E-value cutoff no greater than 10^−5^ and a HMMER parameter E-value no greater than 10^−10^. As a result, a total of 22,680 Unigenes with annotated information were obtained, as shown in Table 2. A total of 6,013 Unigenes were aligned to the COG database, accounting for 26.51% of All-unigenes; 13,147 Unigenes were aligned to the GO database (57.97% of All-unigenes); 9,699 Unigenes were aligned to the KEGG database (42.76% of All-unigenes); 13,568 Unigenes were aligned to the KOG database (59.82% of All-unigenes); 16,551 Unigenes were aligned to the Swiss-Prot database (72.98% of All-unigenes); and 22,217 Unigenes were aligned to the nr database (97.96% of All-unigenes).

**Table 2:** Unigene annotation table.

Annotated databases	Unigene	≥ 300 nt	≥ 1,000 nt
COG	6,013	6,013	4,598
GO	13,147	13,147	9,723
KEGG	9,699	9,699	6,666
KOG	13,568	13,568	9,455
Swiss-Prot	16,551	16,551	11,371
nr	22,217	22,217	13,634
All	22,680	22,680	13,704

Values indicate the overall number of annotated Unigenes the number of those longer than 300 bases (≥ 300 nt), and those longer than 1,000 bases (≥ 1,000 nt) in each of the listed functional databases. COG, clusters of orthologous groups; GO, gene ontology; KEGG, Kyoto Encyclopedia of Genes and Genomes; KOG, EuKaryotic Orthologous Groups; nr, National Center for Biotechnology Information.

#### Functional classification by GO, COG

**Fig 2:**
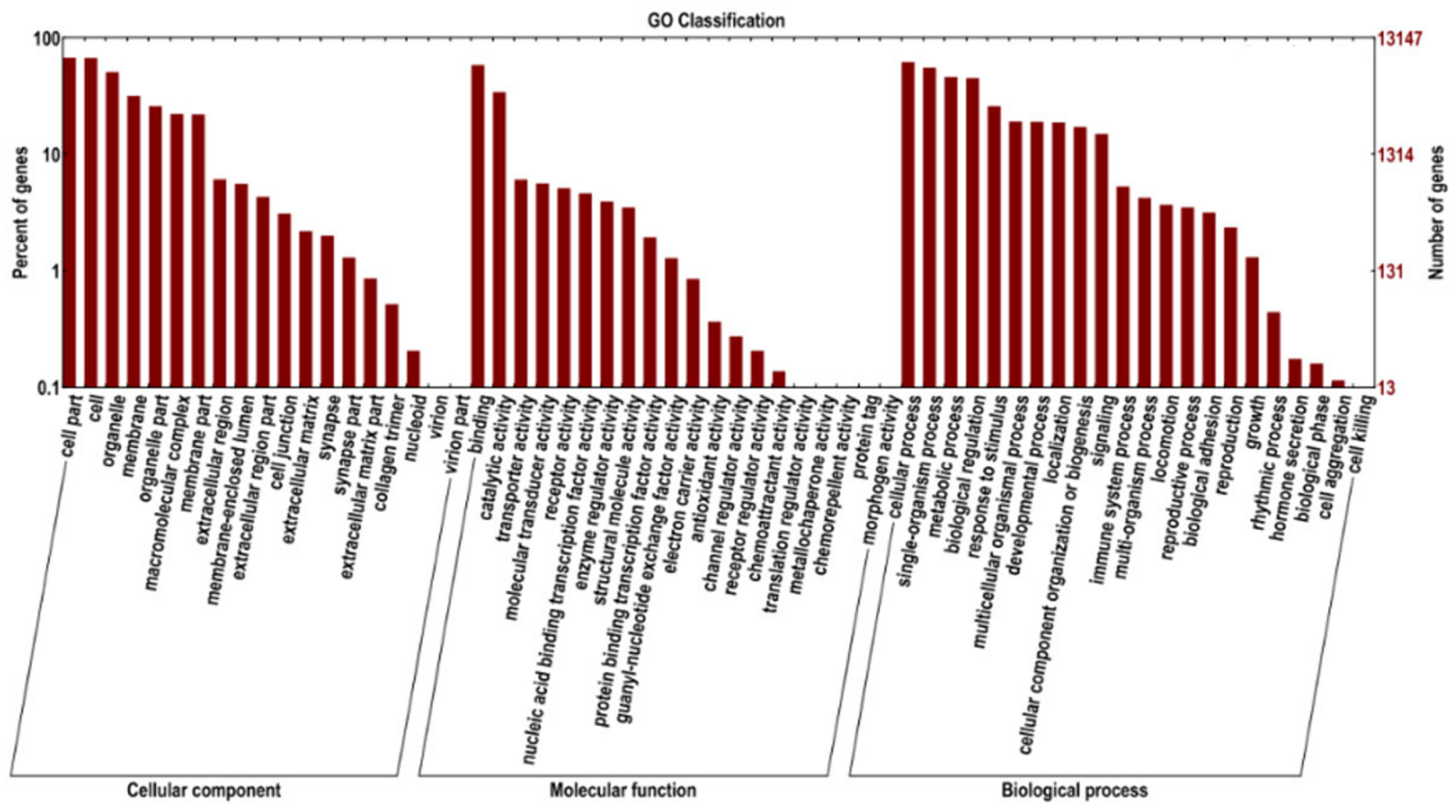
GO functional classification. Secondary nodes under the three main GO categories (X-axis) versus number and percentage of annotated genes at a given node (Y-axis).

Using the GO functional classification, 13,147 All-unigenes were classified into 61 functional categories (Fig 2). Of these, 16,677 genes were classified as Molecular Function, 41,271 as Cellular Component, and 45,783 as Biological Process. Among all GO functional subclasses, 8,848 genes were classified as Cell, making this the largest group; whereas the least represented group was Morphogen Activity, with only two genes.

**Fig 3:**
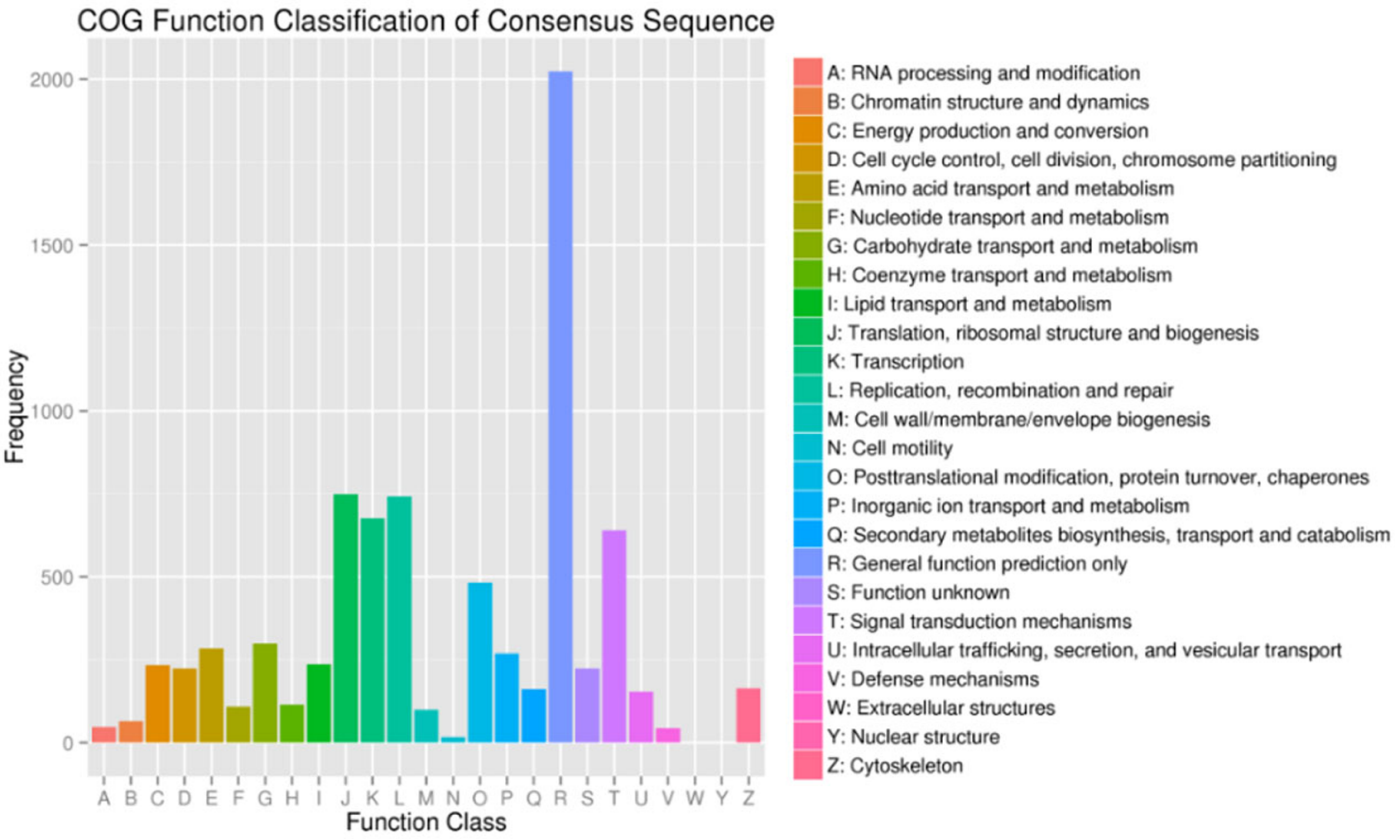
COG functional classification. COG classes (X-axis) versus gene frequency (Y-axis). The number of genes in different functional classes reflects the metabolic and physiological bias in a certain period or environment.

Using the COG functional classification, a total of 6,013 All-unigenes were classified into 25 functional categories (Fig 3). A total of 2,053 genes were classified as General Function Prediction Only, the most numerous of all categories.

#### KEGG pathway analysis and KEGG annotation of differentially expressed genes

**Fig 4:**
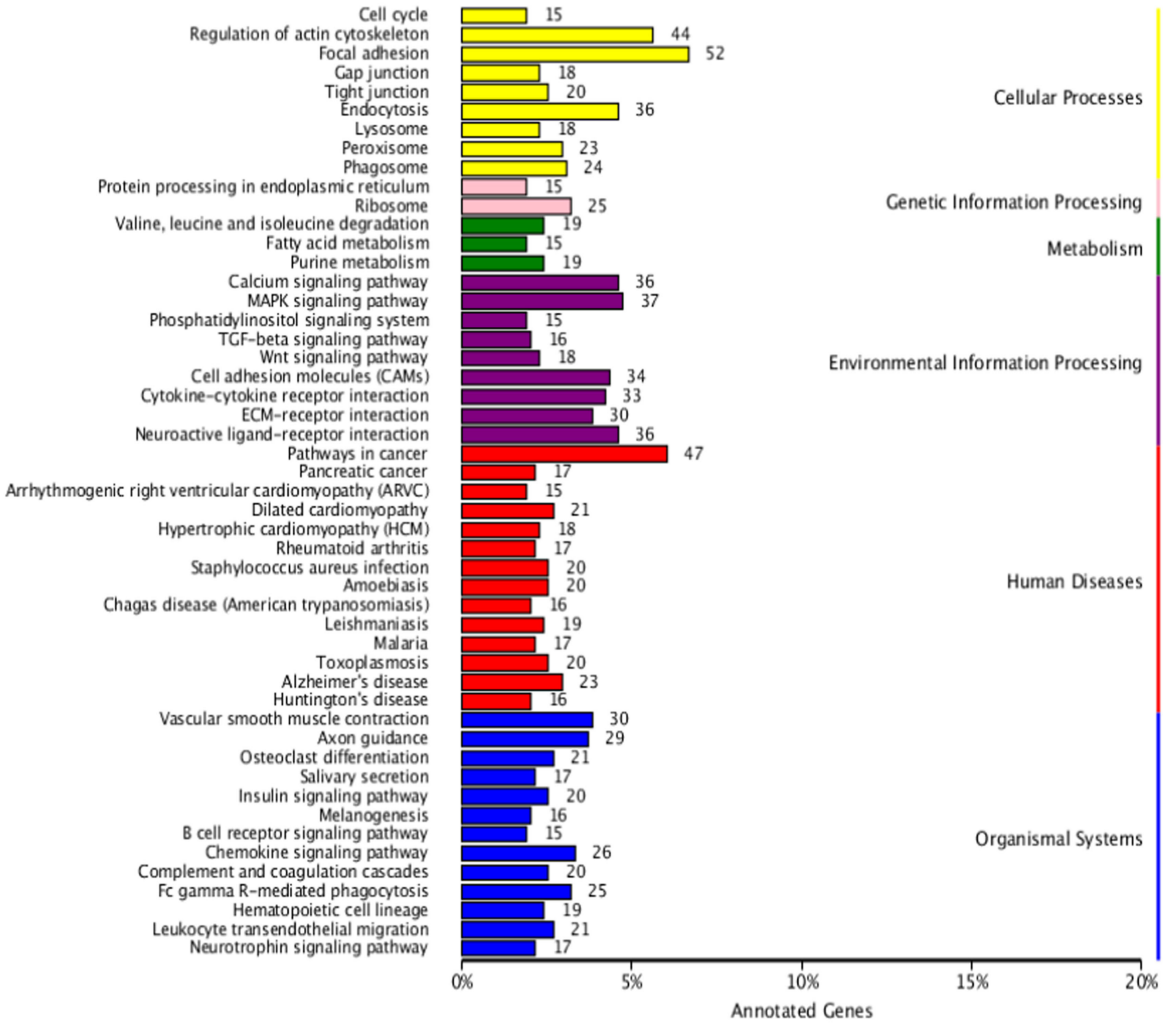
KEGG classification of differentially expressed genes. Percentage of annotated genes (X-axis) versus KEGG categories (Y-axis).

**Fig 5:**
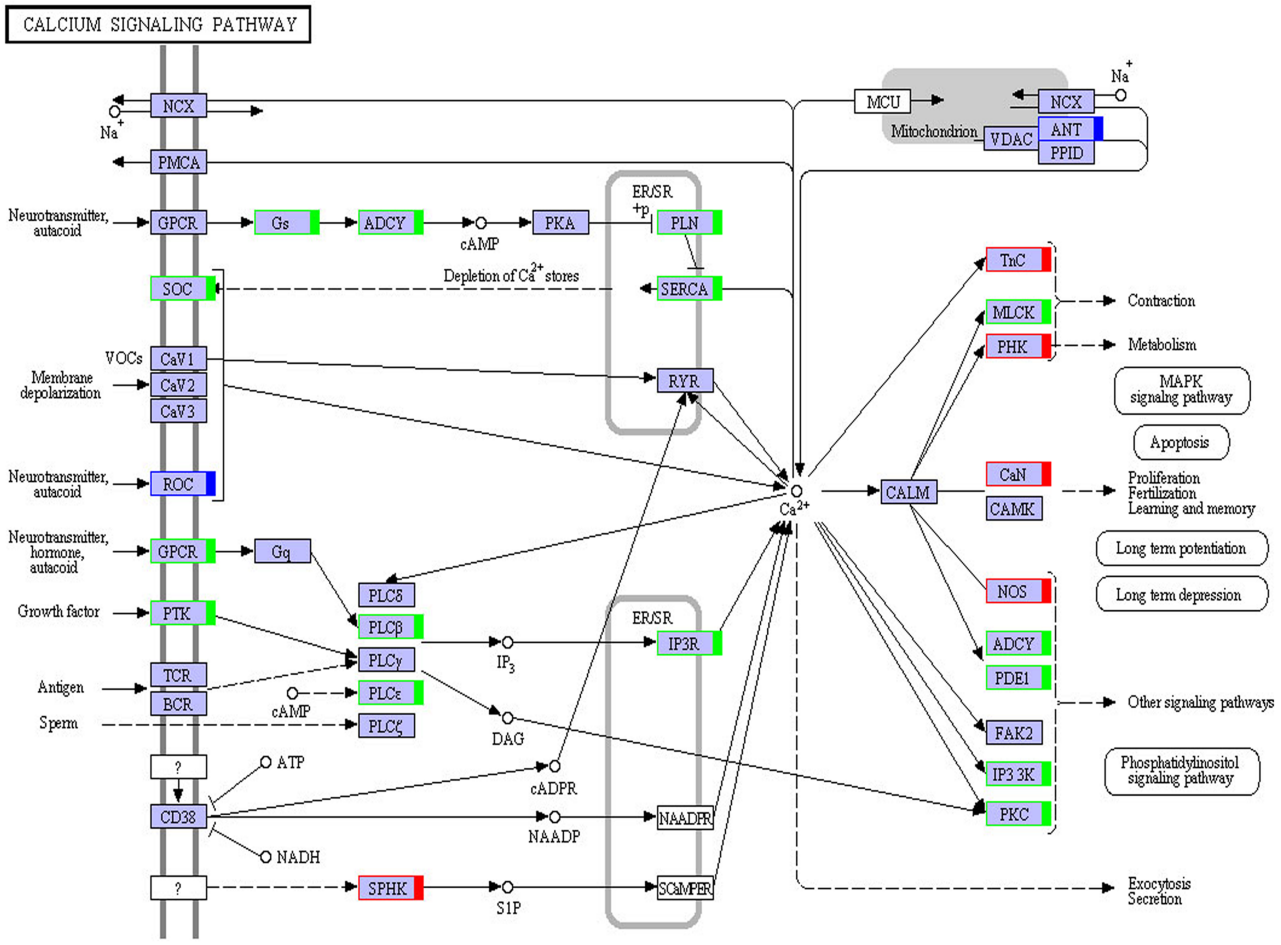
Calcium signalling pathway. Red boxes, genes up-regulated in the musk secreting season; green boxes, genes down-regulated in the musk secreting season; blue boxes, genes that were both up- and down-regulated.

**Fig 6:**
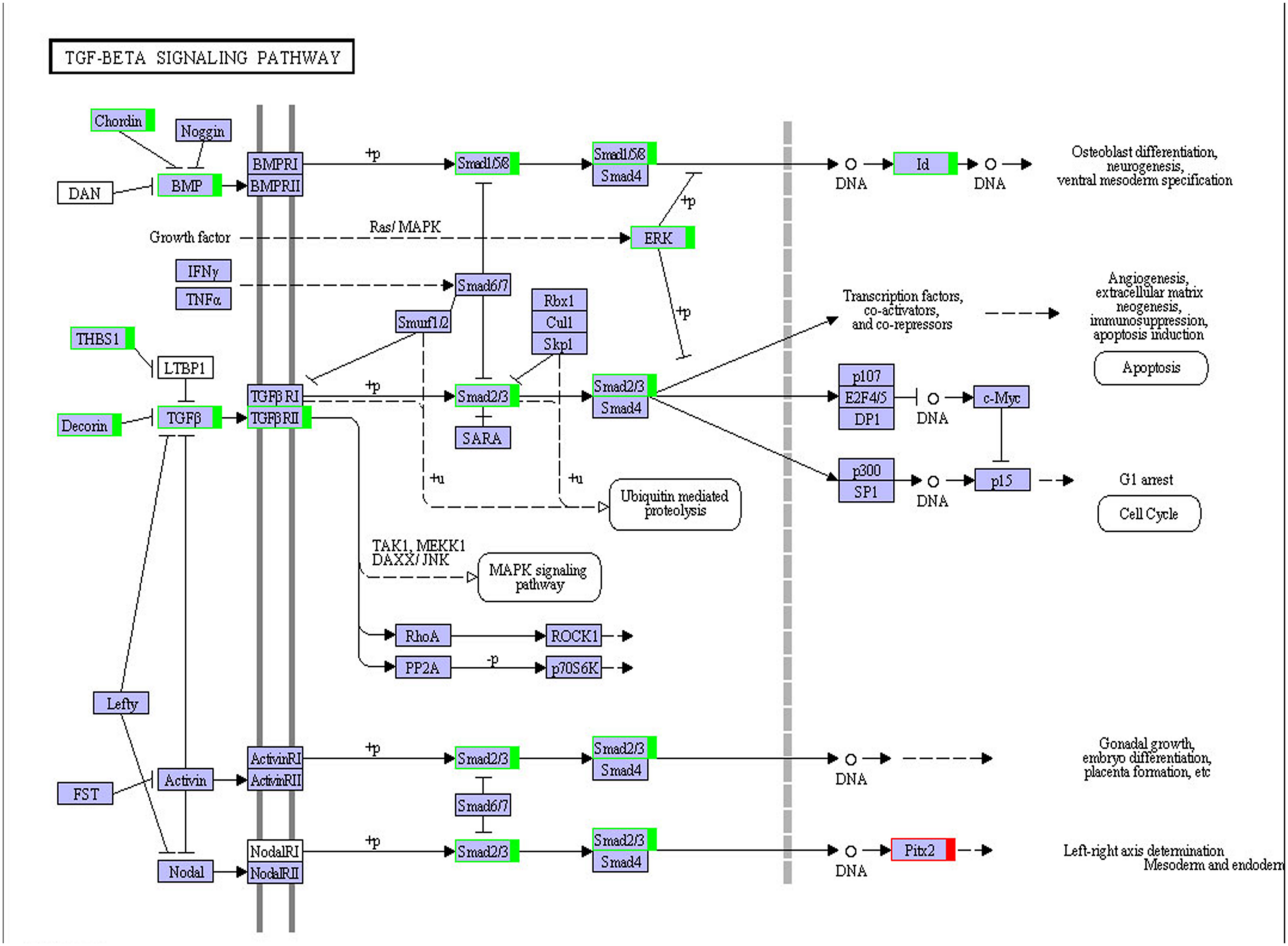
TGF-beta signalling pathway. Red boxes, genes up-regulated in the musk secreting season. Green boxes, genes down-regulated in the musk secreting season; blue boxes, genes that were both up- and down-regulated.

**Fig 7:**
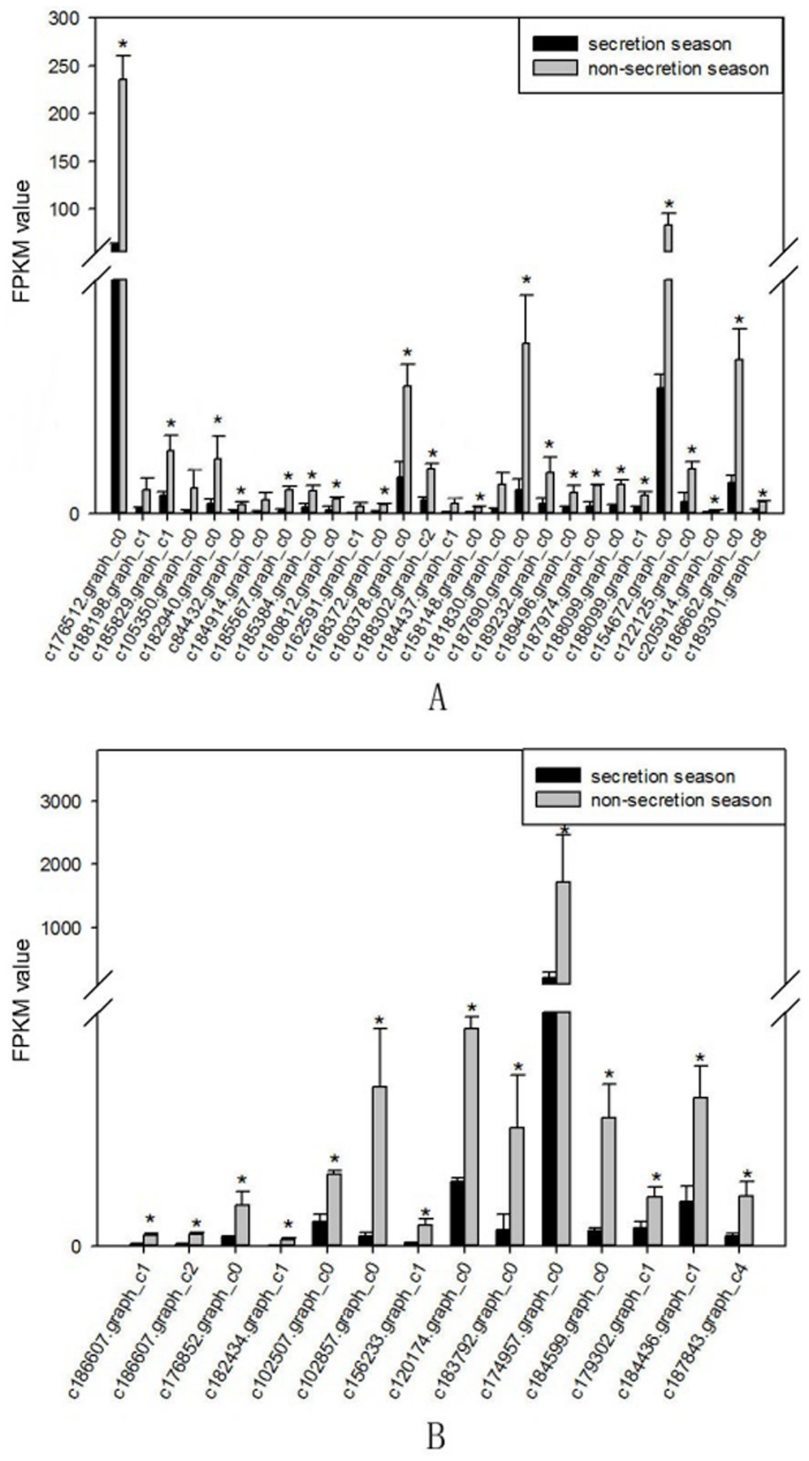
Accumulation of muskrat scented gland cell apoptosis-related transcripts involved in musk production during secreting season and non-secreting season. A. Calcium signalling pathway. B. TGF-beta signalling pathway. Gene IDs are indicated. Data are presented as means + SD (n = 6). *FDR<0.01 and Fold change≥2. FDR, False Discovery Rate. FPKM, fragments per kilobase of transcript per million mapped reads.

A total of 9,699 genes were aligned into the 277 signalling pathways of the KEGG database. The classification is shown in Fig 4. The differentially expressed genes were annotated into six main metabolic categories: Cellular Processes, Environmental Information Processing, Genetic Information Processing, Human Diseases, Metabolism, and Organismal Systems. Annotation results of differentially expressed genes in the calcium signalling pathway (ko04020) and the TGF-beta signalling pathway (ko04350) are shown in Figs 5 and 6, respectively. Genes whose expression was up-regulated in the musk secreting season were closely related to cell proliferation. Down-regulated genes during the secreting season exhibited higher expression in the non-secreting season. The differentially expressed genes identified by transcriptome are listed in S2 File. The most relevant changes to the expression of genes involved in muskrat scented glands degeneration signalling pathways are listed in Tables 3 and 4. The accumulation of the two signalling pathway related transcripts involved in musk production during secreting and non-secreting season is detailed in Fig 7.

**Table 3:** Calcium signalling pathway (ko04020)-related changed genes.

Unigene ID	Name	Regulated	Log_2_FC
c176512.graph_c0	GNAS complex locus	Down	-1.7494
c188198.graph_c1	Adenylatecyclase type 3	Down	-2.02247
c185829.graph_c1	short transient receptor potential channel 1	Down	-1.61826
c105350.graph_c0	cardiac phospholamban	Down	-3.87722
c182940.graph_c0	sarcoplasmic/endoplasmic reticulum calcium ATPase 3 isoform 2	Down	-2.30061
c84432.graph_c0	vasopressin V1a receptor	Down	-1.88038
c184914.graph_c0	prostaglandin E2 receptor EP3 subtype	Down	-3.55149
c185567.graph_c0	cysteinyl leukotriene receptor 1	Down	-2.97074
c185384.graph_c0	type-1 angiotensin II receptor isoform X1	Down	-1.78273
c180812.graph_c0	histamine H1 receptor isoform X1	Down	-1.94352
c162591.graph_c1	substance-P receptor	Down	-4.54254
c168372.graph_c0	thromboxane A2 receptor	Down	-2.39237
c180378.graph_c0	proteinase-activated receptor 1	Down	-1.70397
c188302.graph_c2	leukotriene B4 receptor 2	Down	-1.56667
c184437.graph_c1	CHRNA7-FAM7A fusion protein isoform X2	Down	-3.09987
c158148.graph_c0	glutamate receptor ionotropic, NMDA 2D	Down	-2.3844
c181830.graph_c0	P2X purinoceptor 1	Down	-2.55489
c187690.graph_c0	platelet-derived growth factor receptor beta isoform X1	Down	-2.71805
c189232.graph_c0	1-phosphatidylinositol 4,5-bisphosphate phosphodiesterase beta-4 isoform X1	Down	-1.93084
c189496.graph_c0	1-phosphatidylinositol 4,5-bisphosphate phosphodiesterase beta-2 isoform X3	Down	-1.90381
c187974.graph_c0	1-phosphatidylinositol 4,5-bisphosphate phosphodiesterase epsilon-1 isoform X3	Down	-1.80746
c188099.graph_c0	inositol 1,4,5-trisphosphate receptor type 1	Down	-1.89922
c188099.graph_c1	inositol 1,4,5-trisphosphate receptor type 1 isoform X3	Down	-1.67501
c154672.graph_c0	ADP/ATP translocase 1	Down	-1.63913
c122125.graph_c0	myosin light chain kinase, smooth muscle isoform X4	Down	-1.91967
c205914.graph_c0	calcium/calmodulin-dependent 3′,5′-cyclic nucleotide phosphodiesterase 1A isoform X3	Down	-2.04641
c186662.graph_c0	inositol-trisphosphate 3-kinase B	Down	-2.195
c189301.graph_c8	protein kinase C beta type isoform X2	Down	-1.91075

FC: Fold change is a measure describing how much a quantity changes going from an initial to a final value; Log_2_FC: Expresses the FC of multiple values.

**Table 4:** TGF-beta signalling pathway (ko04350)-related changed genes.

Unigene ID	Name	Regulated	Log_2_FC
c186607.graph_c1	TPA: chordin-like	Down	-2.63768
c186607.graph_c2	chordin	Down	-2.49099
c176852.graph_c0	bone morphogenetic protein 7	Down	-2.07302
c182434.graph_c1	mothers against decapentaplegic homolog 9	Down	-4.22736
c102507.graph_c0	DNA-binding protein inhibitor ID-1	Down	-1.4185
c102857.graph_c0	DNA-binding protein inhibitor ID-3	Down	-3.86741
c156233.graph_c1	DNA-binding protein inhibitor ID-4	Down	-2.56585
c120174.graph_c0	mitogen-activated protein kinase 3 isoform X1	Down	-1.58742
c183792.graph_c0	thrombospondin-2	Down	-2.83224
c174957.graph_c0	decorin precursor	Down	-2.84541
c184599.graph_c0	transforming growth factor beta-1 isoform X2	Down	-2.9319
c179302.graph_c1	transforming growth factor beta-3	Down	-1.31498
c184436.graph_c1	TGF-beta receptor type-2 isoform X1	Down	-1.58897
c187843.graph_c4	mothers against decapentaplegic homolog 3 isoform X3	Down	-2.22258

FC: Fold change is a measure describing how much a quantity changes going from an initial to a final value; Log_2_FC: Expresses the FC of multiple values.

### RT-PCR analysis

**Fig 8:**
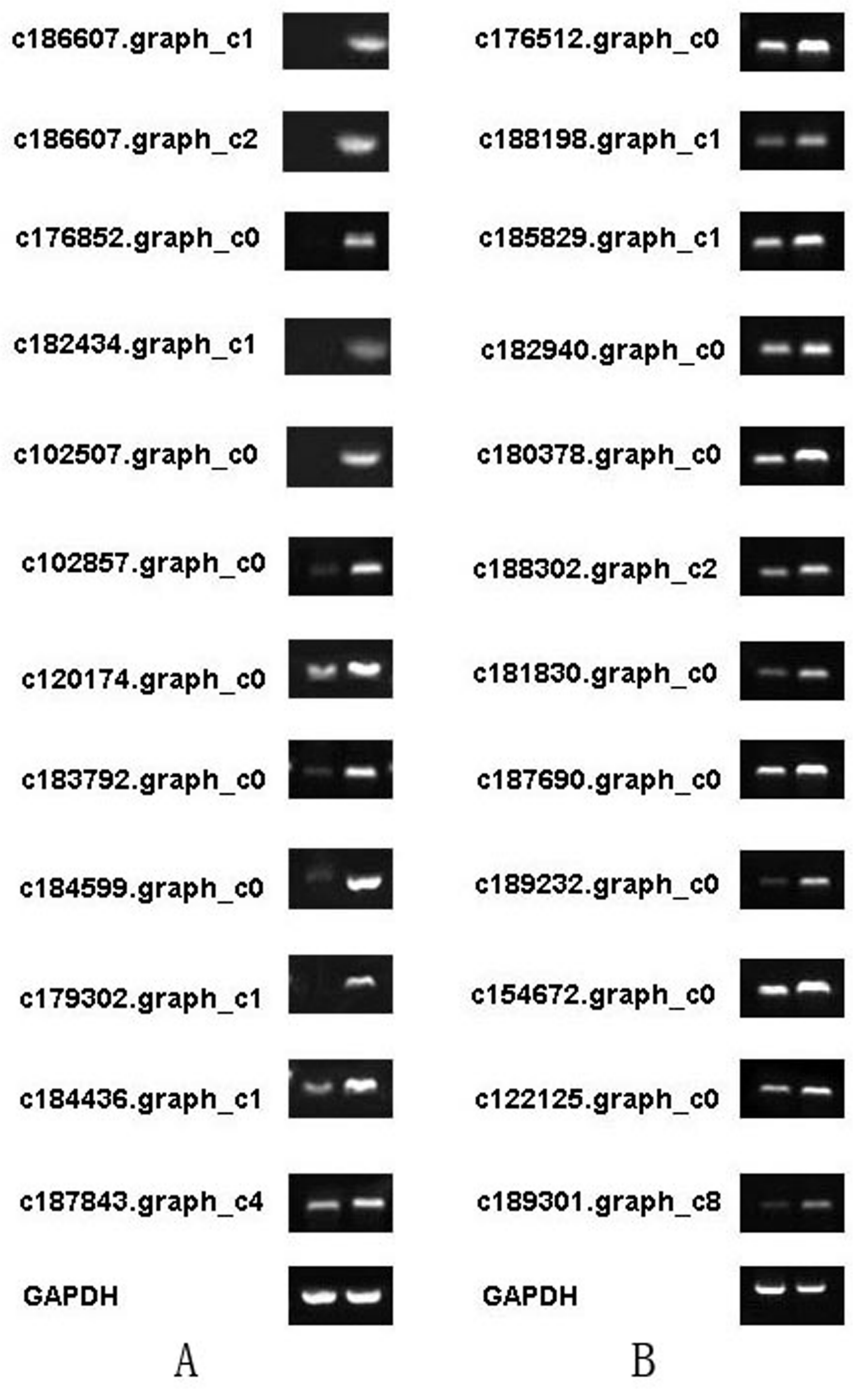
Total cDNA amplified by RT-PCR from scented gland cell apoptosis-related genes. A Alanine, aspartate, and glutamate metabolism. B Glycine, serine, and threonine metabolism. Gene IDs are indicated. Each gene is shown in two columns: secretion season (left) and non-secretion season (right). GAPDH was used as internal control.

**Fig 9:**
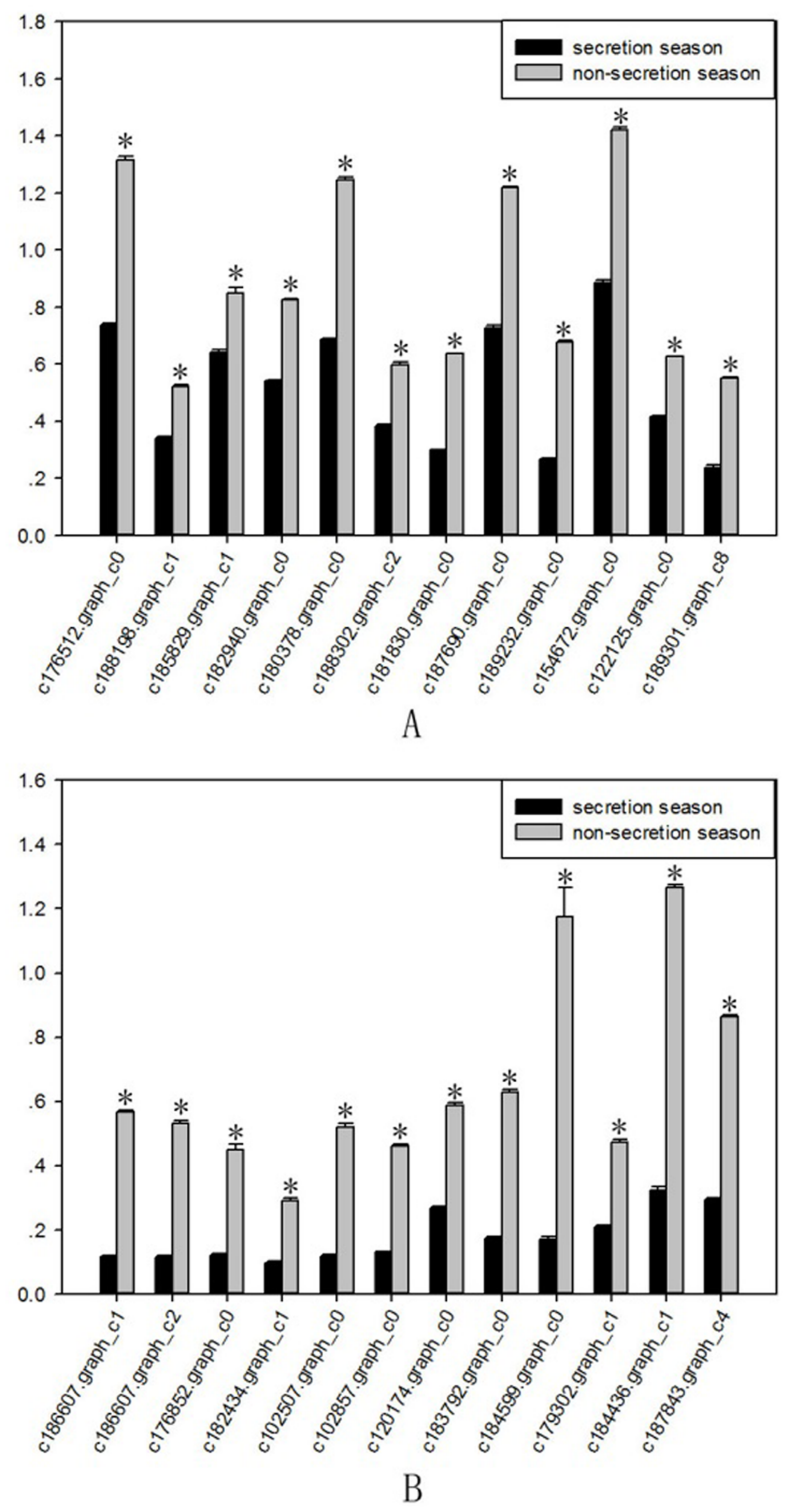
RT-PCR quantification of muskrat scented gland cell apoptosis-related genes during secretion and non-secretion season. A Alanine, aspartate, and glutamate metabolism. B Glycine, serine, and threonine metabolism. Gene IDs are indicated. Data are presented as means + SD (n = 6). *P < 0.05 (Student’s *t*-test).

Based on transcriptome analysis results, we used reverse transcription PCR (RT-PCR) to validate the expression of muskrat scented gland degeneration-related genes. RT-PCR results showed altered expression of genes from the Calcium signalling pathway (ko04020) and TGF-beta signalling pathway (ko04035) in muskrat scented glands. These changes were consistent with the results obtained by transcriptome analysis, which prove that our analysis results reflect the true expression level of scented gland degeneration-related genes. The results of RT-PCR were shown in Fig 8, and the quantification histograms were shown in Fig 9. It should be noted that several genes were not detected by RT-PCR due to very low expression levels.

## Discussion

This study applied Illumina Hiseq 2500 high-throughput sequencing technology to study gene expression of muskrat scented gland tissue from secreting and non-secreting seasons. By employing the *de novo* assembly strategy to assemble short sequence reads, we obtained a total of 57,799 Unigenes with high assembly integrity, which were suitable for gene functional analysis. Analysis of Pearson correlation coefficients between two samples in the same condition showed elevated correlation among specimens from the same season, suggesting that transcriptome analysis results were highly reliable. By aligning to nr, Swiss-Prot, GO, COG, and KEGG databases, information on gene annotation, functional classification, and metabolic pathways was obtained.

COG is an early database for the classification of orthologous gene products generated by a vast number of comparisons of protein sequences from various organisms. Several genes were found to belong to the General Function Prediction Only; Translation, Ribosomal Structure and Biogenesis; and Replication, Recombination and Repair COG classes. This finding suggests that during growth and development of scented glands, genes involved in ribosome formation, protein synthesis, and DNA replication and repair played a key regulatory role.

The GO database provides an international standard for gene functional classification, a comprehensive description of genes, and functional attributes of gene products for an organism. It encompasses three main categories: Molecular Function, Cell Component, and Biological Processes. Each describes the possible molecular function of a gene product, cellular environment, and the biological processes involved. According to the GO classification alignment, a greater number of genes fell in one of the following subclasses: Cell, Cellular Process, Metabolic Process, Cell Component, Binding, and Catalytic Activity. This finding indicates that growth and development of scented glands are closely associated with cell structure formation, cell metabolism, and enzyme-catalysed reactions.

The KEGG database is the primary public repository devoted specifically to pathways. In this study, a large number of genes were annotated in the KEGG Metabolic, Focal Adhesion, Amoebiasis, Regulation of Actin Cytoskeleton, and Extracellular Matrix (ECM)-receptor interaction pathways. This suggests that genes involved in cell metabolism, cytoskeleton interaction, ECM-receptor interaction, and immunity played an important regulatory role during growth and development of scented glands. Interestingly, several genes were also annotated in the Cancer pathway. This should not be surprising given that, during the glands rapid growth period, the fast proliferation of glandular cells is similar to that of malignant tumours. Such phase is thus referred to as cancer-like growth by some researchers.

The gradual degeneration of muskrat scented glands from the secreting to the non-secreting season requires close regulation of cell apoptosis by cytokines. This study found that in the TGF-beta and calcium signalling pathways most genes were significantly more expressed in the non-secreting than in the secreting season. Changes in gene expression showed a significant correlation with alterations in scented gland size. RT-PCR of differentially expressed genes confirmed the above results. TGF-beta is a multifunctional protein which plays important role in inhibiting cell proliferation, regulation of cell phenotype, tumorigenesis and apoptosis of tumor cells [27], bone formation and reconstruction [28], immune-modulating[29], regulation of reproductive function[30–31] and so on. A large number of in vitro and in vivo experiments showed that TGF-beta could induce apoptosis in many kinds of cells [32–35]. For example, TGF-beta can induce growth arrest and apoptosis of lymphocyte in human or mouse [36]; In addition, Liao et al [37] found that TGF- beta 1 play a role in inducing apoptosis of hepatocytes. Smads are a newly discovered family of proteins and part of the intracellular signal transduction cascade elicited by TGF-beta [38]. In particular, Smad2/3 phosphorylation is a key step and a marker denoting activation of the Smads signal transduction pathway. Accordingly, phosphorylated-Smad2/3 levels reflect the extent of activation of the Smads signalling pathway [39]. As shown in Fig 6, smad2/3 expression was higher in the non-secreting than in the secreting season, and hence the extent of Smads signalling pathway activation was higher in non-secreting season. This observation confirms a role for TGF-beta in regulating muskrat scented glandular cell apoptosis in non-secreting season. In addition, we speculated that TGF-beta may also affect the reproductive function of muskrat (musk secreting season corresponding to breeding season, musk non-secretion season corresponding to non-breeding season), this hypothesis need to be further studied. In terms of calcium signalling, a variety of cellular functions are regulated by it, such as muscle contraction, neurotransmitter, release of hormones and enzymes, lymphocyte activation and proliferation [40]. Furthermore, the three apoptotic pathways mentioned before (mitochondrial, death receptor, and ER) involve free calcium. Their ultimate goal is to activate the apoptosis effector caspase-3, resulting in degradation of various cellular components and eventually cell death. Studies have shown that changes in cytoplasmic calcium concentration regulate a variety of cell activities. The response of cells to elevated free calcium levels is dependent on the duration and intensity of the calcium signal: a high concentration of free calcium for an extended period of time promotes cell apoptosis [41]. As shown in Fig 5, calcium-related genes were up-regulated during the non-secreting season, suggesting that increased calcium concentration led to muskrat scented gland degeneration. Normal degeneration of scented glands is associated with developmental changes and scent-secreting performance. Apoptosis during the non-secreting season may help maintain the continuous renewal and dynamic balance of glandular cells. Cell apoptosis in scented glands during the non-secreting season is a complicated physiological process controlled by multiple factors and, so far, studies on possible regulatory mechanisms are still scarce.

Taken together, our results show that the Calcium and TGF-beta signalling pathways play a role in musk gland degeneration. This study comprehensively analysed the characteristics of the muskrat scented gland transcriptome, provided genomic data for future research on muskrat musk biology, and supported further understanding of the seasonal changes and scent-producing function of muskrat scented glands.

## Supporting information

S1 File PCR primers used for qRT-PCR validation. (XLSX)

S2 File Differentially expressed genes identified by transcriptome comparison between secretion season and non-secretion season. (XLS)
